# Polymeric Denture Base Materials: A Review

**DOI:** 10.3390/polym15153258

**Published:** 2023-07-31

**Authors:** Ahmed Yaseen Alqutaibi, Abdulmajeed Baik, Sarah A. Almuzaini, Ahmed E. Farghal, Ahmad Abdulkareem Alnazzawi, Sary Borzangy, Afaf Noman Aboalrejal, Mohammed Hosny AbdElaziz, Ihab Ismail Mahmoud, Muhammad Sohail Zafar

**Affiliations:** 1 Department of Substitutive Science, College of Dentistry, Taibah University, Al Madinah 41311, Saudi Arabia; afrgal@taibahu.edu.sa (A.E.F.); anazawi@taibahu.edu.sa (A.A.A.); sbarzanji@taibahu.edu.sa (S.B.); mhosseiny@taibahu.edu.sa (M.H.A.); 2 Prosthodontics Department, College of Dentistry, Ibb University, Ibb 70270, Yemen; 3 College of Dentistry, Taibah University, Al Madinah 41311, Saudi Arabia; tu3900585@taibahu.edu.sa (A.B.);; 4 Oral Biology Department, College of Dentistry, Ibb University, Ibb 70270, Yemen; afafaboalrejal@gmail.com; 5 Fixed Prosthodontics Department, Faculty of Dental Medicine, Al-Azhar University, Cairo 11884, Egypt; 6 Removable Prosthodontics Department, Faculty of Dental Medicine, Al-Azhar University, Cairo 11884, Egypt; ehababdalhay.9@azhar.edu.eg; 7 Department of Restorative Dentistry, College of Dentistry, Taibah University, Al Madinah 41311, Saudi Arabia; 8 Department of Dental Materials, Islamic International Dental College, Riphah International University, Islamabad 44000, Pakistan

**Keywords:** denture base material, polymers, prosthesis

## Abstract

An ideal denture base must have good physical and mechanical properties, biocompatibility, and esthetic properties. Various polymeric materials have been used to construct denture bases. Polymethyl methacrylate (PMMA) is the most used biomaterial for dentures fabrication due to its favorable properties, which include ease of processing and pigmenting, sufficient mechanical properties, economy, and low toxicity. This article aimed to comprehensively review the current knowledge about denture base materials (DBMs) types, properties, modifications, applications, and construction methods. We searched for articles about denture base materials in PubMed, Scopus, and Embase. Journals covering topics including dental materials, prosthodontics, and restorative dentistry were also combed through. Denture base material variations, types, qualities, applications, and fabrication research published in English were considered. Although PMMA has several benefits and gained popularity as a denture base material, it has certain limitations and cannot be classified as an ideal biomaterial for fabricating dental prostheses. Accordingly, several studies have been performed to enhance the physical and mechanical properties of PMMA by chemical modifications and mechanical reinforcement using fibers, nanofillers, and hybrid materials. This review aimed to update the current knowledge about DBMs’ types, properties, applications, and recent developments. There is a need for specific research to improve their biological properties due to patient and dental staff adverse reactions to possibly harmful substances produced during their manufacturing and use.

## 1. Introduction

The denture base is a part of the denture that carries artificial teeth and rests on the soft tissues in the oral cavity. Denture base materials (DBMs) are particular biomaterials used for fabricating the denture base. To perform in a complex and dynamic oral environment, an ideal DBM should have a wide range of mechanical, physical, chemical, and biological properties, which include strength and durability, processing precision and dimensional stability, acceptable thermal characteristics, biocompatibility, high insolubility and low sorption in oral fluids, chemical stability, excellent aesthetics, ease of production and cleansing, adhesion to artificial teeth, cost-effectiveness, and color stability [1]. Biologically, DBMs should be biocompatible, non-irritant, non-toxic, and non-carcinogenic. Chemically, DBMs must be insoluble, non-absorbable, non-reactive, compatible, and bonded to artificial teeth and denture liners [2]. Mechanically, DBMs should have a high elastic modulus, proportional limit, resilience, adequate abrasion resistance, fatigue, and impact strength. Physically, DBMs should have low specific gravity, dimensional stability, good thermal conductivity, radiopaquicity, a coefficient of thermal expansion matching to teeth, and thermal stability. Esthetically, DBMs need to be translucent and have the ability to pigments to match the color of teeth and gums. In addition, there are favorable properties such as low cost, ease of manipulation and repair, ease of cleaning, and long shelf life [2,3,4,5].

Although a dental implant is increasingly used in treating partial and completely edentulous patients, conventional partial and complete dentures are still the treatment of choice in many cases due to financial and medical issues. However, currently, no DBM has all the ideal physical and mechanical properties, biocompatibility, and esthetic properties. Each DBM has its own limitations. For example, polymeric DBMs are prone to fracture, have bad insulators, and have low density. On the other hand, metallic DBMs are rigid, good conductors of heat, but heavy due to high density, costly, and require a technique-sensitive casting procedure. An attempt to improve one property is likely to compromise any other property. Therefore, developing a DBM with all the ideal properties is very challenging. Researchers have recently investigated various DBMs, introducing a wide range of modifications with promising outcomes [6]. Therefore, this comprehensive review aimed to update the current knowledge about DBMs’ types, properties, applications, and construction methods. In addition, various recent developments in the field of DBMs were discussed.

The dental industry is continuously evolving, and new materials and techniques are being developed to improve the properties and performance of denture bases. Therefore, this review is essential for dental professionals, researchers, and students who want to stay up-to-date with the latest advances in denture base materials and their applications. Additionally, this review will provide insights into the limitations of current denture base materials and the potential for future developments. Overall, this review will serve as a valuable resource for anyone interested in denture base materials, providing a deeper understanding of their properties, applications, and recent advancements.

## 2. Materials and Methods

A thorough literature search on denture base materials was conducted using electronic databases, including PubMed, Scopus, and Embase, in October 2022. The final search was carried out in May 2023 to ensure the inclusion of updated articles on DBMs. Additionally, websites dedicated to journals in the fields of dental materials, prosthodontics, and restorative dentistry were looked up. A manual search was carried out by looking through the articles’ references. The following keywords were used for the search: ((“denture base materials” OR “denture base” OR “PMMA” OR “polymethyl methacrylate” OR “denture resin”) AND (“properties” OR “modification” OR “application” OR “construction” OR “biocompatibility” OR “toxicity” OR “patient reaction” OR “manufacturing” OR “recent developments”)).

Studies were chosen if they had been written in English and had information on the modifications, different types, characteristics, applications, and construction methods concerning denture base materials.

## 3. Evolution/History of DBMs

The concept of using DBMs existed centuries ago. Before the 17th century, denture bases were fabricated using natural materials such as wood, ivory, and the bones of hippopotamuses or whales that were carved to fit the spaces in edentulous regions. In the 18th century, Etienne Bourdet first used gold for making denture bases, but their widespread use in dentistry was prevented due to high cost and poor esthetics due to their color [7]. De Chemant prepared the first set of porcelain dentures; however, their widespread use was limited because of brittleness, heavy weight, and lack of natural appearance. In 1839, vulcanized rubber was discovered by Charles Goodyear. Vulcanite’s introduction as a DBM resulted in a significant decrease in the dentures’ cost. Vulcanite successfully replaced previous BDMs owing to its appropriate properties such as comfort, economy, ease of preparation, lightweight, and dimensional stability. However, its long-term use was restricted because its properties were still far from ideal, such as poor esthetic and lack of chemical bonding with the porcelain teeth [7]. In 1867, Bean made a casting of the first denture base fabricated from aluminum alloy. Additionally, in 1888, Carroll introduced a method of casting aluminum dentures under pressure. Although the accuracy and weight of these dentures were acceptable, their use was discouraged because of the difficulty in relining and the high fabrication cost. In 1870, celluloid was introduced. One of its favorable properties was the ability to be stained pink to match the gingiva and oral mucosa color. However, its popularity soon faded because of changing its pink color due to the absorption of stains from drinks and food. Moreover, patients complained about camphor residual taste. In 1909, Leo Bakeland introduced Bakelite. Despite the material possessing excellent esthetics, its frequent use for denture fabrication was prevented due to its tendency for staining, inherent brittleness, and difficulty of fabrication and repair [7]. In 1930, polyvinyl chloride was used for denture fabrication, which received insufficient popularity because of its inherent mechanical weakness and potential to discolor following exposure to hot food and liquids. In 1937, base metal alloys were used for the fabrication of dentures. As a result of their lightweight properties, strength, and low price, these materials became a popular choice for fabricating dentures [7]. Despite this, these materials were hard to repair, could cause allergic and cytotoxic reactions, and were corroded and tarnished, resulting in poor esthetics. In 1937, Walter Wright first introduced Poly (methyl methacrylate) (PMMA) as DBM. By 1946, this material became one of the most used materials for denture fabrication due to its favorable properties, which included ease of processing and pigmenting, sufficient mechanical properties, economy, and low toxicity. Despite the wide use of PMMA, its physical and mechanical properties do not meet the requirements of an ideal DBM. This is because of its susceptibility to fracture under cyclic loading and water absorption, which can adversely affect its mechanical properties [1,8].

## 4. Applications of Denture Base Materials

**Figure 1:**
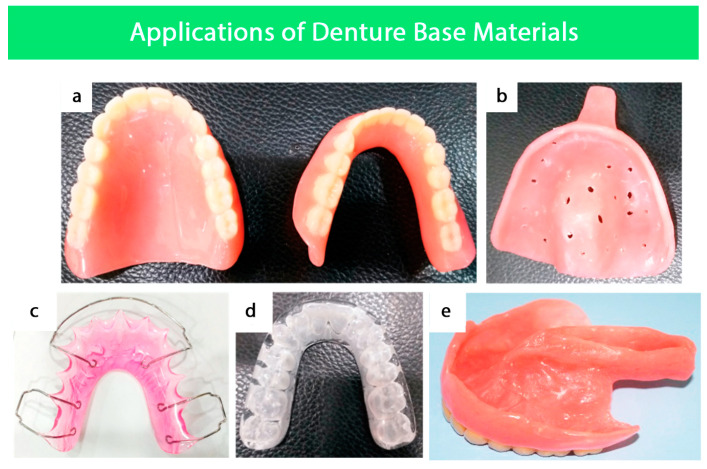
Examples of applications of acrylic materials in dentistry: (**a**) complete denture; (**b**) secondary impression tray; (**c**) orthodontic retainer; (**d**) occlusal splint; (**e**) palatal obturator replacing lost tissue following maxillectomy [2].

In addition to denture bases, the DBMs are also used for various other applications in dentistry (Figure 1), which are discussed in this section.

### 4.1. Fabrication of Removable Partial and Complete Dentures

Removable complete dentures are fabricated conventionally with acrylic resin. Despite cobalt chromium partial dentures having many advantages in various clinical situations, an all-acrylic removable partial denture is frequently used [9]. PMMA is widely utilized because of its exceptional physical and mechanical qualities in the manufacturing of removable dentures. PMMA is used in every stage of denture production, from creating customized trays to finished appliances. In addition, PMMA is also used for the special tray that takes the final impression of the edentulous arch because of its high dimensional stability and reduced handling forces [9].

The PMMA trial denture base is used to verify the precision of the impression. Before moving on to the last step, the dentist can examine the denture’s fit, stability, and occlusion. PMMA is also utilized for the acrylic teeth in the denture since it is adaptable to different sizes and shapes. PMMA is used for the final denture because of its high strength, transparency, and biocompatibility. The acrylic teeth are fastened firmly to the PMMA denture base, which is molded to the patient’s mouth. The denture is then polished to a shiny, lifelike finish [2,9].

### 4.2. Surgical Splints

Surgical splints are used in orthognathic surgery as tooth-borne positioning guides to translate surgical plans into clinical practice. Simulated surgical movements are conventionally performed on dental casts to plan for the surgery. The splints are created by combining pre-polymerized PMMA powder with a liquid component containing MMA monomers and a cross-linking agent such as ethylene glycol dimethacrylate. These splints serve as guides during the surgery to ensure the surgical plan is executed accurately [10,11].

### 4.3. Secondary Impression Trays

Heat-cured, self-cured, and light-cured acrylic resins are frequently used to fabricate special trays for secondary impressions. Compared to heat-cured PMMA, cold-cured PMMA has some drawbacks, including lower strength and the possibility of amine accelerator oxidation, which can result in poor performance. The light-cured PMMA is used to create special trays but it is typically unsuitable for use as a DBM [12].

### 4.4. Orthodontics

PMMA is used for the fabrication of multiple orthodontic appliances, including retainers, bite guards, myofunctional appliances, occlusal splints, and bite planes. The material’s characteristics and processes for orthodontic appliance fabrication are similar to those for denture bases made of PMMA; however, they differ in design and functional capacities [2].

### 4.5. Obturators

An obturator is a special prosthetic device that is used to restore missing maxillary tissues and functions, including mastication, deglutition, speech, and esthetic. The material most used for obturators is PMMA. The injection molding technique is used to overcome polymerization shrinkage since it provides better accuracy and marginal sealing than conventional compression molding. However, there are some drawbacks related to PMMA obturators, for example, polymerization shrinkage if a conventional molding technique is used, in addition to difficulty with undercuts due to the rigidity of the material or pressure sores in delicate tissues [2].

## 5. Desired Properties of DBMs

### 5.1. Physical Properties

#### 5.1.1. Sorption and Solubility

The process by which materials absorb water while immersed is called sorption [1]. Solubility can be defined as the maximum amount of a solute that can dissolve in a solvent for a given period at a given temperature [1]. According to ISO 20795-1, the sorption should be less than 32 µg/mm^3^, while solubility should be less than 1.6 µg/mm^3^ [13]. The sorption and solubility of currently available PMMA DBMs are far below the ISO 20795-1 requirements [14,15]. However, heat-cured PMMA materials have lower solubility and sorption than cold-cured PMMA materials [2].

#### 5.1.2. Thermal Conductivity

Thermal conductivity is defined as the heat flow rate per unit temperature gradient [9]. DBMs must have adequate thermal conductivity to conduct the food temperature to the oral tissues [2]. Metallic denture bases have high thermal conductivity compared to PMMA. The thermal conductivity of PMMA is low (5.7 × 10^−4^ C/Cm); for that reason, the heat generated during the denture fabrication cannot escape and cause surface crazing [2,5]. Additionally, the low conductivity can affect the ability of the patient to sense the food temperature; consequently, extremely hot drinks may reach the pharynx or esophagus without having any sensation and may burn the delicate soft tissues [1,2,5].

#### 5.1.3. Color Stability

Ideally, DBMs must have high color stability in a complex oral environment. However, PMMA has poor color stability due to multiple factors [2]. A high residual monomer content and a poor degree of conversion lead to reduced color stability [1]. In addition, fabrication porosity and frequent beverage consumption, such as alcohol, tea, and coffee, are associated with color changes and staining [2].

#### 5.1.4. Polymerization Shrinkage

Polymerization shrinkage produces significant dimensional changes and inaccuracies in denture fabrication. The lowest polymerization shrinkage level is required for dental applications [2]. According to Kopperud et al., light-cured PMMA leached a significantly greater amount of MMA compared to thermoplastic PMMA and powder-and-liquid-based PMMA [16]. Regarding heat-cured PMMA, the injection molding technique exhibited less polymerization shrinkage and improved the marginal seal compared to conventional compression molding. Continuous injection of the PMMA in the injection molding technique compensates for the polymerization shrinkage. In addition, modifications of PMMA, for instance, reinforcement by adding fibers or carbon nanotubes, can significantly decrease the polymerization shrinkage and increase the dimensional accuracy of dental prostheses [2].

#### 5.1.5. Radiopacity

The ideal DBM is required to be radiopaque so that it appears white on diagnostic radiographs and can be easily distinguished from tissue, such as if a fractured piece is swallowed accidentally [1,2]. Inherently, PMMA is a radiolucent material that is difficult to distinguish in radiographs [2]. To improve the radiopacity, heavy metals were incorporated or metallic DBMs were used alternatively [2]. Although incorporating various heavy metals has enhanced the radiopacity to a certain degree, there are some concerns, for example, the inability to physically or chemically bind additives to the matrix and leaching of salts out of the denture base [1,2]. According to Lang et al., incorporating triphenyl bismuth (30% *w*/*w*) into PMMA enhanced the radiopacity without disrupting the esthetic and mechanical properties [17].

### 5.2. Mechanical Properties

Good mechanical properties are required for DBMs to withstand the complex functional and masticatory forces in the oral cavity [2].

#### 5.2.1. Flexural Strength

Flexural strength is defined as the measure of the strength of a bar under a static load that is supported on either end by lower supports [1]. Based on ISO 20795-1 (2013) for DBM, flexural strength is measured by a three-point bending test [13]. Flexural strength is a combination of compressive, tensile, and shear stresses. It measures the denture base resistance against fracture caused by bending [1]. Ideally, denture bases must have a high flexural strength to withstand the complex forces of mastication without fracture or permanent deformation [2]. According to Barbosa et al., the flexural strength of cold-cured, heat-cured, and microwave-cured PMMA is good (84.40 ± 1.68, 92.84 ± 4.73, and 109.63 ± 5.31 MPa, respectively) [18]. However, the flexural strength of PMMA-based dentures is influenced by several factors, including chemical composition, curing method, degree of polymerization, dimensions, and storage [2].

#### 5.2.2. Fracture Toughness

The ability of a material to resist crack propagation from notches or defects on its surface is known as fracture toughness [2]. According to ISO 20795-1 (2013), three-point bending of DBM specimens with a notch in the midline is used to test fracture toughness [13]. The fracture toughness of heat-cured PMMA (2.06 ± 0.17 MN/m^3/2^) is significantly greater than cold-cured PMMA (1.63 ± 0.1 MN/m^3/2^) [18]. The fracture toughness of DBM can be increased by long-term immersion [1].

#### 5.2.3. Impact Strength

Impact strength represents the amount of energy required to fracture a denture base under the influence of an impact force. High impact strength is required to resist denture fracture when subjected to a high-impact force like accidental dropping [2,19]. Robinson and McCabe (1993) reported that the impact strength of PMMA DBMs significantly reduced in the presence of surface defects as small as 16 μm [20]. By adding butadiene styrene rubber to PMMA, the impact strength can be considerably enhanced, but other properties may be reduced, such as hardness and modulus of elasticity [1,2].

#### 5.2.4. Surface Hardness

Resistance of a material to plastic deformation is typically measured under an indentation load [3]. A surface’s hardness directly affects its wear resistance. Compared to casting alloy and dental porcelain, PMMA wear resistance is significantly low [2,21,22,23]. The surface hardness of heat-cured PMMA is more significant than cold-cured PMMA.

### 5.3. Biological Properties

A material’s biocompatibility is its capability to perform in biological environments and to create a favorable response from the host [1,2]. An essential requirement of DBMs is to be safe and nontoxic in the oral cavity. DBM cytotoxicity depends on the chemical nature of the DBM, the polymer/monomer ratio used, the degree of residual monomer, and the polymerization method. DBM can significantly reduce cytotoxicity by immersing the denture in water before use and extending the polymerization time [1]. Microwave-cured and heat-cured PMMA exhibited lower monomers and cytotoxicity compared to cold-cured PMMA [2].

#### 5.3.1. Biocompatibility of Acrylic Resins

An essential concern surrounding the clinical use of polymeric DBMs is biodegradation. It may be described as the alterations in their physical, mechanical, and chemical characteristics due to oral environmental conditions [1]. In the mouth, the materials are more prone to complicated involving diverse endogenous compounds (polysaccharides, bacteria, proteins, enzymes) and exogenous compounds (types of components that are delivered from dairy consumption diet) (all types of compounds from the dairy intake diet). These components produce complicated interactions, resulting in a significant mechanical action in a general biodegradation event towards the biomaterials in the oral cavity. These operations could change the material’s characteristics and jeopardize performance [24]. Furthermore, the biodegradation of a biomaterial might result in leachable compounds, which can trigger a cascade of biological reactions in tissues and cells. Although the influence of biodegradation on the biocompatibility of acrylic materials is debatable, there is growing worried regarding its clinical implications as objective and subjective complaints related to these materials increase [4,24]. Polymeric materials have traditionally been characterized as huge stable structures with excellent biodegradability. Several investigations have demonstrated that polymers may be susceptible to various biodegradation mechanisms in the oral cavity. Polymer breakdown happens due to various causes, including pH and temperature fluctuations, salivary enzymes, chemical and nutritional changes, and chewing, encouraging their biodegradation [25,26,27,28,29,30,31,32].

##### Consequences of Biodegradation

The spread of potentially hazardous uncured/unbound monomers or/and additives from the polymer network is a crucial clinically relevant impact of PMMA biodegradation. The released chemicals may harm oral tissues, such as local irritation, systemic toxicity, and allergic response. Biodegradation and stability may cause significant changes in a material’s mechanical and physical characteristics, potentially leading to catastrophic collapse [25,26,33,34,35,36,37,38,39,40].

##### Release of Compounds from Acrylic-Based Resins

A lot of attention has been paid to the chemicals that are released from DBMs. Under experimental conditions, polymer specimens of various forms (cylinders, rectangles, disks) and sizes are generally incubated in a liquid at room temperature according to the manufacturer’s instructions; the duration can range from an hour to two months [24,41,42,43,44,45,46,47,48,49,50,51,52,53]. Water is often utilized as the leaching medium in most research. Ethanol and ethanol/water combinations have improved the solubility of insoluble-water chemicals such as phthalates. It has been reported that residual monomers and other leachable components migrate from acrylic-based products into human and artificial saliva. The spread of MMA from auto-polymerized and heat-cured acrylic resins was evaluated using unstimulated whole human saliva. It was discovered that artificial saliva made of an esterase enzyme and an aqueous buffer improved the rate of phthalate diffusion from a soft-lining acrylic resin substance [24,45,46,51,52,53,54,55,56,57,58,59].

An in vivo and in vitro investigation was conducted to assess the spread of phthalates from two soft denture lining materials based on polyethyl methacrylate polymers. The findings showed that in vivo plasticizer loss was more significant than in vitro plasticizer loss. Phthalates were found in salivary samples taken from individuals who wore dentures or orthodontic gear. Under in vivo settings, significant levels of MMA and formaldehyde were detected in human saliva leaking from acrylic auto-polymerized resins [47,57,60,61]. Phthalates are a group of chemical compounds that have been widely used in the production of soft relining materials for dentures. However, research has shown that phthalates can pose health risks to humans, including potential hormonal disruptions and adverse effects on reproductive health, so fewer soft-relining materials contain phthalates [62].

The amounts of residual MMA monomer in auto-polymerized acrylic resin were recently measured in forty volunteers. High residual monomer concentrations were found during the first twenty-four hours of usage. The elution of unbound components, including additives such as benzoyl peroxide, phthalate esters, and MMA monomer, is cited in most studies as one of the primary outcomes of material biodegradation [6,24,26,41,53,55,56,58,63,64]. During the first few hours of polymerization, additives and unbound monomers are eluted, and their release depends on the time.

Generally, the most residual monomers are released in the first twenty-four hours following processing, followed by a moderate and steady release over time. The residual monomer was found in dentures worn for up to seventeen years, with the bulk of this release occurring in the first five [56,59,65,66,67]. The chemical mechanisms involved in releasing chemicals from acrylic-based resins have been studied. pH’s effect on biodegradation has been studied, and it was shown that salivary acidity had a significant impact on the leaching characteristics of denture base acrylic resins if the pH is decreased, indicating larger quantities of MMA monomer [55,56,68]. A large quantity of formaldehyde was discovered to be generated as an oxidation product of the leftover MMA monomer leaking from auto-polymerized acrylic resins. Methacrylic acid, a byproduct of MMA hydrolysis, has been observed seeping out of acrylic soft lining materials and acrylic denture base in artificial saliva, water, and ethanol–water solutions. It has also been discovered that benzoic acid is produced from the benzoyl peroxide initiator [51,55,62,66,69,70].

##### Biological Effects of the Release

Products delivered from the biodegradation process of acrylic resins are suspected to be responsible for labial edema, ulceration, discomfort in oral tissue, sensitization, chemical irritation, and other oral illnesses such as burning mouth syndrome and denture stomatitis. Farid et al. (2022) reported that residual monomers can cause allergic reactions and contribute to denture stomatitis, a fungal infection of the oral mucosa [41]. The study also notes that although some types of residual monomer may have bactericidal properties, this may not be sufficient to prevent the colonization of microorganisms on the surface of dentures over the longer term. Hence, it is essential for denture wearers to be aware of the potential risks associated with residual monomer and to follow proper oral hygiene practices to prevent the development of infections or other adverse reactions.

Aromatic carboxylic acid esters and phthalates used in relining acrylic materials as plasticizers could have negative health consequences [46,54,62,66,71]. Allergic responses to acrylic resins are linked to MMA monomers and additives. According to cell culture methods, chemicals that spread from acrylic resins will produce various biological reactions in cells [70,72,73,74]. This paper cannot cover all articles that assess the impact of acrylic-based resins on cells. The cytotoxicity of leached MMA monomer and its derivatives has been investigated. In the experiments, both permanent (osteoblast and L 929 fibroblast) and primary cells, such as epithelial, dental pulp, gingival fibroblast, and periodontal ligament cells, are employed. Cytotoxicity is tested in different ways; all show alterations in fundamental cell structures, such as cell membrane integrity, and cell functions, such as enzyme activity or macromolecule production [50,56,73,75,76].

The mechanism of MMA monomer toxicity is likely to entail direct toxicity from residual MMA and oxidative stress generated by free radicals released during resin polymerization. Recently, gene expression analysis has been utilized to assess the influence of MMA on the expression of antioxidant enzymes such as glutathione. Cell culture methods show that released MMA monomer in acrylic resin biomaterials could induce genotoxicity and changes in growth factor expression and cell cytokine. Other methacrylate monomers, such as isobutyl methacrylate and 1,6 hexanediol dimethacrylate, which are key components in numerous relining acrylic polymers, have been studied in several investigations. The cytotoxicity impact of these monomers was shown to be on periodontal cells and fibroblasts in a dose-dependent manner [50,75,77,78,79,80]. The pH value influenced the leaching of cytotoxic components and adverse effects. Changes in the mechanical and physical characteristics of PMMA are susceptible to various intraoral occurring circumstances, which might affect their structural integrity or dimensions. Biodegradation may harm the material’s internal qualities and affect the binding strength between reline acrylic resins and denture bases [68,69,70].

Polymeric denture materials can degrade over time due to various factors such as mechanical stress, oral pH, and exposure to saliva. Degradation can result in the release of particles and chemicals from the material, which can potentially cause toxicity concerns. The degradation of denture base materials can lead to the release of toxic substances such as formaldehyde, methyl methacrylate, and benzene. These substances can cause adverse health effects such as irritation, sensitization, and even cancer. Furthermore, the degradation of denture base materials can also lead to the release of nanoparticles, which can potentially cause toxicity concerns [41].

##### Inner Properties of PMMA

Most previous investigations have focused on the effects of water absorption on materials’ dimensional changes. Its effects on acrylic polymers’ mechanical and physical characteristics, such as resistance to plastic deformation, hardness, fatigue limit, and flexural strength, have been investigated. Water molecules may seep inside the gaps between the polymer chains and push them apart. Consequently, the secondary chemical bonding forces between the polymer chains weaken, causing weight and volume to rise and expand. The larger the material’s water absorption, the more significant the related dimensional change [80,81,82,83,84,85]. Water molecules may serve as plasticizers over time, changing in mechanical characteristics of the polymer. If the water molecules have a plasticizing impact on the resins, the mechanical characteristics should decrease following water immersion. The various effects of water immersion on the mechanical characteristics of the resins might be attributed to differences in their chemistry. In terms of strength, if the ingredient that leaches out has a deeper plasticizing impact than water molecules, the denture polymer’s strength should rise. In contrast, if the ingredient that leaches out has a lower plasticizing impact than water molecules, the denture polymer’s strength may drop [80,84,86,87].

Other factors, such as resin type, thickness, and number of cross-linked polymers, can influence the degree of dimensional changes caused by water absorption. Because of its lower water diffusion coefficient, a heat-polymerized denture acrylic polymer requires more time to attain saturation than an auto-polymerized polymer [86,87]. The viscoelastic characteristics of the materials may also be influenced by chewing. It has been shown that cyclic loading influences the viscoelastic characteristics of acrylic-based soft lining materials, particularly delayed deformation. Reduced delayed deformation suggested that soft lining materials had lower stress distribution effects.

Furthermore, it was proposed that following cyclic loading, water absorption per surface area would rise [87,88]. Acrylic resin DBMs exhibited clinical variations in viscoelasticity by a faster and greater decrease in compliance than in vitro media, such as artificial saliva, water, or denture cleaners. A solvent impact caused by food changes might explain these disparities. Solvents are mixtures of ethanol and water that function as food-mimicking liquids. The hydrophilic monomer matrix absorbs large quantities of water molecules and ethanol. The solvent enters the resin matrix, facilitating its softening and shattering [89,90].

#### 5.3.2. Antimicrobial Modification of PMMA

Although PMMA is a commonly used material for denture base construction, it may have certain limitations due to its lack of antimicrobial properties. As a result, there is a growing interest in developing PMMA modifications that can provide antimicrobial properties. One strategy involves incorporating antimicrobial agents, such as silver nanoparticles or quaternary ammonium compounds, into the PMMA matrix [41]. Another approach is to modify the surface of the PMMA with antimicrobial coatings or grafting techniques. These modifications aim to prevent the growth and colonization of bacteria and fungi on the denture surface, which can lead to oral infections and other health complications [3].

Studies suggest that fluoride glass fillers [91,92], fluorapatite, and apatite-coated TiO_2_ [93,94] are efficacious in averting microbial adherence and proliferation on PMMA surfaces. It has been observed that the antibacterial effect of fluoride in the oral cavity is enhanced due to the presence of the oral microbiome [2,95]. Additionally, nanomaterial-based strategies, specifically silver nanoparticles [96,97,98,99,100], nanodiamonds [101], and mesoporous silica nanoparticles loaded with antifungal medication amphotericin B [102] have demonstrated significant inhibition of *Candida albicans* proliferation in the presence of denture stomatitis. Notably, these additives do not compromise the surface and flexural characteristics of PMMA when the thymoquinone antifungal agent is incorporated up to concentrations of 1% [103].

The antibacterial efficacy of quaternary ammonium compounds has been scrutinized extensively [104], and these compounds have been utilized to modify a diverse array of dental materials. When incorporated into restorative dental materials, quaternary ammonium-based compounds display potent antimicrobial activity [105,106]. However, utilization at elevated concentrations could influence the polymerization shrinkage, flexural strength, modulus, and biocompatibility of the materials [104].

Cured PMMA, post-treatment with 2% quaternary ammonium compounds, demonstrated antibacterial and antifungal activity in vitro. Dentures containing quaternary ammonium compounds could potentially avert denture stomatitis in elderly individuals predisposed to the condition [107,108].

Surface functionalization represents an alternate strategy to impart antibacterial properties and prevent bacterial adhesion on a surface [109]. By altering the material surface, engineers can minimize the impact on the material’s bulk properties. PMMA surface functionalization via oxygen plasma and thermal treatment was reported by Mai et al. [110]. Antimicrobial action was incited by incorporating chlorhexidine, which was released from the functionalized surface in a controlled, gradual manner. Furthermore, cytotoxicity assays showed no indications of cellular damage or apoptotic cell death [110].

Lee et al. [111] characterized PMMA concerning its physical and antibacterial properties post-incorporation of graphene oxide nanosheets. Integrating graphene oxide nanosheets and a non-thermal oxygen plasma surface treatment enhanced hydrophilicity and anti-adhesive properties [111]. PMMA has been recently formulated with several food preservatives, such as sodium metabisulfite and potassium sorbate. The modified PMMA materials demonstrated acceptable flexural properties and enhanced antibacterial activity without exhibiting cytotoxicity [112]. Despite the impact on mechanical properties, including food preservatives did not compromise the material’s flexural capabilities.

Most studies that support the integration of antimicrobial nanoparticles have been conducted in vitro. However, the dynamic and complex oral environment, including its antibacterial activity, may influence material responses. Therefore, further in vivo clinical investigations are required to validate the antimicrobial activity of these agents and confirm their biosafety and biocompatibility.

## 6. Classification of DBMs

Denture bases can be classified into either metallic or non-metallic. Metallic materials include gold alloy type IV and cobalt-chromium alloy (Co-Cr), while non-metallic denture base materials include acrylic resin. Furthermore, denture base polymers can be classified based on their processing and packing behavior into the open flask technique as conventional heat-cured acrylic resin and the injectable technique as thermoplastic acrylic resin (Thermopress) [113].

### 6.1. Polymeric DBMs

**Figure 2:**
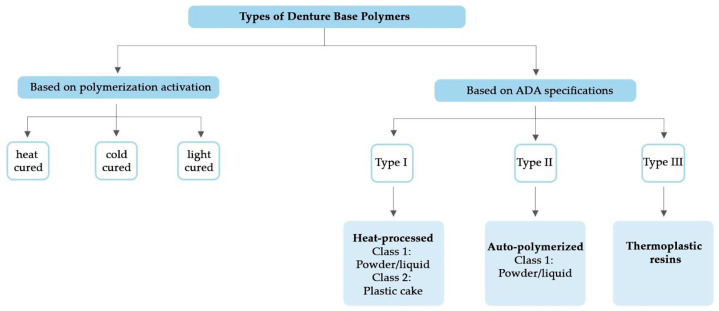
Classification of denture base polymers based on polymerization activation and according to the ADA specifications [2].

Polymers are long-chain molecules composed of many repeating units [9] that can be formed by condensation or addition polymerization [9]. Acrylic resins are polymer esters of methacrylic acids [9]. Because of the superiority of acrylic resin’s characteristics, it has been the most extensively used DBM since its inception in 1937. Other polymers used in small quantities include vinyl styrene, polycarbonates, nylon, ethylene, polyurethane, unsaturated polyesters, polyvinyl acetate [9], polyether ether ketone (PEEK), and polyether ketone ketone (PEKK)) [114,115,116,117]. Denture base polymers are broken down into several categories (Figure 2) based on the ADA’s Specification No. 12. There are three primary types of denture base polymers [11], which may differ from one another in terms of polymerization reaction and content, according on how the polymerization process is activated.

#### 6.1.1. Historical Polymeric DBMs

##### Polycarbonates

Polycarbonates are chains of bisphenol-A carbonate [1]. Although polycarbonates have better mechanical properties than conventional acrylic denture base resins, they are not frequently used for denture fabrication because of complex processing, being expensive, and being prone to toxicity due to the leaching of bisphenol-A (BPA) [1].

##### Acetal

In 1971, acetal was introduced as an unbreakable thermoplastic resin removable partial denture material. The polymerization of formaldehyde produces acetal resins. These resins have high fatigue resistance, biocompatibility, hardness, and low coefficient of friction. However, high fatigue resistance may deleteriously affect the stress resistance and implication of using these resins [9].

##### Polystyrene

Polystyrene was developed and introduced as a DBM in 1948. Polystyrene has high transverse and residual stress. However, due to the lack of flexibility and strength, it had not been consistently used and was discontinued as a denture base material [9].

#### 6.1.2. Acrylic Resins

Acrylic resin is made up of both liquid and powder components. The powder comprises pre-polymerized spheres of polymethyl methacrylate (PMMA) and a small quantity of benzoyl peroxide, “0.5–1%”, that is responsible for the initiation of the polymerization process and is referred to as the initiator, along with pigments, dyes, and opacifiers. The liquid is an unpolymerized methyl methacrylate with a minor quantity of hydroquinone added as an inhibitor to prevent the liquid from polymerizing or setting during storage. The liquid may be cross-linked using a cross-linking agent such as ethylene glycol dimethacrylate (TEGDMA) [3,118]. Polymethyl methacrylate (PMMA) is a transparent glass-like polymer that is sometimes used to produce denture bases. However, manufacturers commonly use pigments to match color with soft tissue. Little fibers coated with colors are occasionally employed to create a veined look. Denture base resins contain mostly iron oxides or titanium oxides as pink pigments. Adding these pigments to a PMMA yielded similar coloration to that of the regular pink PMMA used in dentistry and did not jeopardize its mechanical properties [119,120,121].

According to the way of processing, the acrylic resin denture bases may be further classified into the following categories.

##### Heat-Activated Denture Base Resins

Almost all denture bases are made using heat-activated materials. A microwave oven or water bath can provide the thermal energy needed to polymerize such materials. The majority of heat-activated PMMA resin solutions have both liquid and powder components. The powder comprises pre-polymerized spheres of PMMA and a trace of benzoyl peroxide, which initiates the polymerization process and is referred to as the initiator [16,99]. The liquid is predominantly unpolymerized MMA with a small amount of hydroquinone that acts as an inhibitor to increase the material’s shelf life. A cross-linking agent could be added to the liquid, such as glycol dimethacrylate, usually used in PMMA denture base resin, to increase its strength. Heat-activated denture base resins may also be available as premixed gels formed from polymer and monomer. This gel can be compressed, molded, and packed [17,121].

##### Chemically Activated Denture Base Resin

Chemically activated resins are also known as “self-curing”, “ cold curing”, or “auto polymerizing” resins. The primary difference between self-curing resin and heat-cured resin is that the reaction of polymerization is sped up by a chemical, like N-dihydroxy-ethyl-para-toluidine, instead of heat [18]. Chemically activated resin polymerization is never complete as heat-activated resin, because heat-activated resins typically have 0.2% to 0.5% free monomer, while chemically activated resins have 3% to 5% residual free monomer [122].

##### Pour Type Denture Base Resin

This resin has a chemical composition similar to PMMA that is polymerized at room temperature. The size of the polymer powder or beads is the sole distinction. Pour-type denture resins, or fluid resins, feature much smaller powder particles that, when mixed with the monomer, create a very fluid slurry that can be poured into agar hydrocolloid or modified plaster and polymerized under pressure at 0.14 MPa. To pour the slurry into the mold, centrifugal casting and injection molding are utilized [20].

##### High-Impact Strength Materials

Denture base materials with higher impact strength, such as polymers reinforced with butadiene-styrene rubber, have been introduced in dentistry. The rubber particles can be embedded into methyl methacrylate to allow bonding to the acrylic matrix. These materials are handled like other heat-activated methyl methacrylate materials and delivered in a powder–liquid form. It is indicated for cases with difficulty handling complete dentures, such as very old complete denture wearers, patients with neuromuscular diseases or tremors, and Parkinsonism [121,123].

##### Injection Molding Denture Base Resin

For injection molding materials, waxed dentures are flasked and boiled out similarly to compression molds. A hollow sprue is attached to the wax-created mold cavity in injection molding. An exterior flask opening is coupled to a high-pressure injection cylinder. The base resin for dentures is mixed and located in the cylinder. When the material has reached the desired consistency, it is injected under high pressure into the mold cavity. During the polymerization cycle, the pressure is maintained, and while polymerization shrinkage occurs, additional ingredients enter the flask. It was reported that this type of resin base demonstrated better dimensional accuracy [123,124].

##### Light-Cured Denture Base Resin

A light-cured resin is a composite made up of high molecular weight acrylic resin monomers, microfine silica, and urethane dimethacrylate. As an organic filler, acrylic resin beads are used. The activator is visible light, and the polymerization initiator is camphorquinone [125]. It comes in premixed sheets with a consistency similar to clay. While the denture base material is still malleable, it is adapted to the cast. Without teeth, the denture foundation can be polymerized in a light chamber and utilized as a record base. The teeth are processed with extra material to the base, and their morphology is polymerized in a light chamber with 400–500 nanometer blue light (nm). In the chamber, the denture rotates to offer uniform exposure to the light source [126].

##### Microwavable Resins

Tertiary amines are used to activate these resins. Microwave energy is being used in a new way to cure denture base resins. Curing the resin takes around three minutes in a special fiber-reinforced flask. The cured resin shrank less and absorbed less water [121].

#### 6.1.3. Thermoplastic Resins

Thermoplastic resins are flexible biocompatible materials with unique physical and mechanical properties. These resins were introduced in 1950 to solve many of the restrictions present in traditional acrylic resin because of improved denture adaptability, as well as denture retention due to their light weight and engagement of more desired undercuts. These materials have good esthetics as well as desirable physical properties and ease of fabrication [105,106]. In comparison with powder and liquid resin systems, thermoplastic resins offer several advantages such as better stability, resistance to solvents, high fatigue endurance, and excellent wear properties. The long-term performance of thermoplastic resins is usually predictable. Thermoplastic resins often contain extremely little or nearly no free monomer. These materials provide a novel and safe therapy for people who have an allergy to free monomers. Furthermore, thermoplastic materials have little porosity, reducing biological material buildup, odors, and stains while exhibiting superior dimensional and color stability [127,128,129,130,131]. These considerations come into play when creating long-term interim prostheses for difficult restorative cases or implants or when using permanent, removable appliances. Flexibility and strength can be increased by adding elastomeric resins to resin polymer formulas, resulting in higher flexibility and strength of thermoplastic acrylic resins.

Thermoplastic resins can be reinforced with glass fibers or filler to improve their physical qualities. These restorations have outstanding esthetics and provide the patient with long-term comfort. This provides superior esthetic repair alternatives for patients with esthetic demands [127,129,132]. Thermoplastic resins are utilized in many applications, such as fiber-reinforced fixed partial dentures, removable prostheses, pre-formed partial denture clasps, provisional crowns and bridges, and denture bases. These materials are also used to make occlusal splints, brackets, and orthodontic retainers, speech therapy appliances, sleep apnea appliances, impression tray and border molding materials, implant abutments, and obturators [33,133,134]. The thermoplastic acrylic resin denture base materials can be further classified into the following categories:

##### Thermoplastic Acetal

Thermoplastic acetal exhibits satisfactory mechanical properties in the short term when used as a homopolymer. However, when utilized as a co-polymer, it shows improved long-term stability. Acetal resin is flexible and highly strong simultaneously. It has good fracturing and wear resistance, making it a perfect material for bridges, temporary bridges, single-pressed unilateral partial dentures, occlusal splints, dental implant abutment, and clasps of partial denture [33,128,135].

##### Thermoplastic Polycarbonate

Polycarbonate is a bisphenol-A carbonate polymer chain. It is a commonly used material for prefabricated temporary crowns. Like acetal resin, polycarbonate resin is exceedingly flexible, strong, and resists fracturing. Polycarbonate is appropriate for temporary crowns and bridges; however, not recommended as partial denture frames [123,136,137,138].

##### Thermoplastic Nylon

Nylon is a type of resin produced by combining monomers, dibasic acid, and diamine. It has heat and chemical resistance and high physical strength. Because of its intrinsic flexibility, it is primarily employed in dentistry for flexible tissue-supported removable partial dentures [123,139,140,141].

##### Thermoplastic Acrylic Resin “Versacryl”

This acrylic resin type is known as “bio-compatible intraoral thermoelastic material” and has been utilized in dentistry as provisional crowns and as a denture base material for removable prostheses [123,140,142]. Versacryl (thermoplastic acrylic resin) can be applied for various applications and has sufficient flexural and tensile strength. The material is easy to polish, shape, and manipulate. Additionally, it can be repaired and relined chairside. Versacryl is available in gingival and tooth shades and has great esthetics due to its translucency and vitality. Versacryl, like most thermoplastic resins, is flexible, strong, and resistant to fracturing [143,144]. Versacryl is a heat-sensitive, multi-purpose acrylic with over fifty unique applications for improving removable dental appliances by improving retention, esthetics, and patient comfort. Simply by using warm water, this acrylic may adjust any area of a denture. It can be extended into any undercut, for example, to retain a denture mechanically, to construct repeatable thermoclines, to replace metal clasps in partial dentures, and to create sublingual wings to stabilize lower dentures [143,144].

#### 6.1.4. Modified PMMA

Although PMMA has several benefits, it is not considered an ideal DBM. To enhance the physical and mechanical properties of PMMA, several studies have been performed [2,145,146,147,148,149].

##### Fiber Reinforcement

Different types of fibers have been incorporated to enhance the resins’ fatigue resistance, flexural strength, and impact strength [2,145]. The distinctive advantages of reinforcement are achieved due to the relatively more considerable length of the fiber than its diameter. The morphology of fibers (length, diameter), orientation within the matrix, concentration, pre-impregnation, and silane treatment affect the enhancement of mechanical properties [2,150].

##### Glass Fibers

Research has demonstrated that the incorporation of glass fiber reinforcement leads to a substantial improvement in the impact strength, flexural strength, Vickers hardness, and toughness of acrylic resins; additionally, the deformation of the denture base was significantly reduced to less than 1% [145]. In addition, the flexural properties of the denture base are influenced by the position and orientation of the glass fiber within it. When the glass fiber is situated near the surface of the denture base on its tensile stress side, it enhances the flexural modulus, toughness, and flexural strength [145]. Placing glass fibers in a neutral stress area improved flexural toughness, and placing glass fibers on the compressive side increased surface flexural modulus [145].

##### Polyamides

Polyamides include nylon and aramid fibers [145]. Aramid fibers are biocompatible and have better wettability, increased flexural strength, and flexural moduli [2,145]. However, they have poor esthetics because of their yellow color [2,145]. Additionally, fibers exposed to the surface irritate the tissue, making it difficult to achieve a finished and polished surface [145]. Moreover, the hardness of the resin is reduced with increasing fiber concentration [145]. Nylon fibers can improve structural elasticity, fracture resistance, and flexural strength [2,151].

##### Polyethylene and Polypropylene

Incorporating polyethylene and polypropylene fibers enhanced the impact strength of PMMA, and additional improvement in impact strength was achieved through surface treatment [145,152]. The utilization of a woven polyethylene fiber reinforcement substantially enhances the toughness and elastic modulus of PMMA. However, etching, preparing, and positioning the woven fibers is considered impractical [145]. The introduction of salinized polypropylene fibers to heat-cured PMMA resin resulted in an improvement in transverse, tensile, and impact strength. However, the material’s wear resistance decreased [145,152].

##### Filler Reinforcement

Multiple researchers have examined the use of fillers to enhance the strength of denture base resin. Using metal oxides for PMMA reinforcement improved the material’s mechanical and physical properties; in addition to the patient’s ability to sense hot and cold stimuli, the recent suggestion for improving the properties of PMMA involves the addition of nanofillers. The thermal stability was enhanced, and the thermal properties were improved due to the high surface area, small size, and homogenous distribution of nanofillers. The added particles’ size, type, concentration, and shape affect the resin’s properties reinforced with nano-fillers [145,153,154].

##### Metal Oxides Alumina (Al_2_O_3_)

Adding aluminum to PMMA increased the compressive strength, tensile strength, flexural strength, impact strength, and surface hardness of the resin. Additionally, warpage decreased significantly with adding aluminum to PMMA; water sorption or surface roughness was not significantly affected. Safi (2014) reported that adding Al_2_O_3_ nanoparticles to PMMA increases thermal stability compared to pure PMMA [155]. The incorporation of silanized Al_2_O_3_ nanoparticles into acrylic resin improved the flexural strength and thermal properties of acrylic resin, in addition to decreasing solubility and water sorption [145]. Moreover, Abdulkareem et al. (2015) reported that adding alumina Al_2_O_3_ nanoparticles to microwave-treated and untreated PMMA powder resulted in good biocompatibility [156]. The drawback of aluminum-reinforced PMMA is discoloration, which limits its use in invisible areas [145,157,158].

#### 6.1.5. Other Polymeric Materials

##### PEEK

PEEK is a high-temperature, semi-crystalline material with a high melting temperature [114]. The potential of PEEK as a novel material to substitute PMMA is being explored [116,159,160]. The tensile strength of PEEK specimens that were milled or pressed at a mold temperature of 200 °C was found to be higher. PEEK polymer is regarded as a material with resistance to notch concentration due to its higher Izod impact strength than PMMA [160]. PEEK has the potential to be modified by the addition of other materials. For example, incorporating carbon fibers enhances the elastic modulus of PEEK, increasing it up to 18 GPa. PEEK can be used in computer-aided design and manufacturing systems for creating dentures [114,116].

##### Nylon

Nylon is a term used to refer to specific types of thermoplastic polymers that are classified as polyamides that are created by condensation between a dibasic acid and a diamine [161]. Since the 1950s, nylon has been used as a DBM [139]. PMMA is an amorphous substance, whereas nylon is a crystalline material that exhibits high heat resistance, flexibility, and insolubility in various solvents [82].

Stafford et al. (1986) reported that the impact strength of the nylon denture was significantly greater than that of conventional PMMA [19]. Additionally, the nylon denture exhibited higher flexural strength than PMMA DBMs because of its improved flexibility [162]. The flexural strength of the polyamide denture base was considerably more significant when compared to that of acrylic resin, while the flexural modulus of the nylon DBM was considerably lower than that of PMMA [19].

Polyamide dentures have great resistance against fracture and flexural fatigue because of their excellent flexibility [82]. Therefore, polyamides are beneficial when patients cannot withstand hard denture bases [82]. Furthermore, polyamides can also be utilized in patients allergic to PMMA [82]. However, polyamides are challenging to process and repair [82]. In addition, they have high water sorption, solubility, and potential for staining. For these reasons, polyamides are not used frequently as denture base materials except in a few specific cases [82,163].

### 6.2. Base Metal Alloys (BMA)

Base metal alloys are essential in prosthetic and restorative dentistry [164]. With the advancement of all-ceramic restorations and the creation of more durable resin-based composites, this position has changed dramatically in recent years. However, alloys are still the primary material for most prosthetic restorations and will likely continue to be the primary material for many years. According to American national standard/American dental association specification No. 5, dental casting alloys must fall under one of the following categories: Type 1 dental casting alloys are considered low strength and are suitable for castings that experience minimal stress, such as inlays. Type 2 dental casting alloys are considered medium strength and are appropriate for castings that experience moderate stress, such as onlays and inlays. Type 3 dental casting alloys are categorized as high strength and are intended for castings subjected to high stress levels, such as saddles, thin cast backings, full crowns, pontics, short-span fixed partial dentures, and thick veneer crowns. Type 4 dental casting alloys are classified as extra high strength. They are designed for castings that are subjected to extremely high levels of stress and have thin cross sections, such as clasps, unit castings, saddles, thimbles, bars, thin veneer crowns, removable partial denture frameworks, and long-span fixed partial dentures.

BMA has the necessary wear resistance, strength, modulus, and biocompatibility to function as a prosthesis in the oral cavity [77]. The alloy’s biocompatibility, cost, and physical properties may influence the dentist’s selection for a different clinical scenario. In fixed and removable prosthodontics, many alloys are adopted. Although high noble metal alloys have excellent clinical applications, as the cost of gold has risen, other less expensive-based alloys have been created. However, these BMAs may contain nickel, cobalt, and chromium and can occasionally produce sensitivity. On the other hand, titanium and its alloys demonstrate good biocompatibility for dental applications [143]. Titanium frameworks offer several advantages over traditional dental alloys, such as excellent corrosion resistance [45,46], suitable mechanical properties [47,48], lightweight, improved fitting accuracy [49], and reduced incidence of metals related allergy due to their superior biocompatibility [50,51]. However, forming a chemical reaction layer during the titanium casting process is an unavoidable drawback [52,53].

Despite the long history of using ceramics and alloys as removable and fixed restorative materials, there are still unanswered questions about their biological activity, so research related to prosthodontics should include molecular and cell biology techniques to analyze the host’s immunological, chronic inflammatory, and non-immune responses to materials that come in touch with oral tissues to study this possibility [165,166]. Cobalt chromium (Co-Cr) alloys are commonly applied to fabricate and rehabilitate removable prostheses [167]. Cobalt-based alloys are gaining popularity in the field of dentistry because they combine features such as biocompatibility, wear resistance, and high mechanical properties (hardness, ultimate tensile strength, and yielding strength) [168,169].

Significant variation in dental alloys has been seen over time in manufacturing technologies. In the field of dental technology, Co-Cr alloys, which have ISO 22674 (2006) criteria, Type 4, are intended for dentures with small sections and are subjected to significant forces, and Type 5 for dentures requiring a high degree of mechanical resistance and rigidity, such as complete and partial dentures. The disparity between these two types can be attributed to the increased conventional elasticity limit observed in Type 5. (360 towards 500) [170,171,172].

## 7. Method of Construction

### 7.1. Conventional Method

#### 7.1.1. Resin Denture Base

There are multiple processing techniques for the fabrication of denture bases, all of which necessitate the creation of an accurate impression to generate a dental cast. Next, a resin record base is created on the cast, and wax is applied to the record base to set the teeth. The teeth arrangement is enclosed in a suitable investing medium within a denture flask. The wax is removed after opening the flask, and the mold is cleaned. A resin denture base material is injected into the mold cavity, and the denture base resin is subsequently polymerized [3].

#### 7.1.2. Metal Denture Base

The lost wax casting method is employed to create metal denture bases. After taking an impression and pouring a cast, a wax model is produced in the desired shape. The model is then placed in an investment material that can withstand high temperatures. The wax is subsequently melted and burned away, leaving a cavity in the desired form behind. Finally, the cavity can be filled with molten metal [5].

### 7.2. Computer-Aided Design and Manufacturing (CAD/CAM) and Rapid Prototyping

CAD/CAM technology pertains to digital design and manufacturing [173] and involves subtractive techniques such as milling [174]. The denture base is milled from preformed acrylic resin blocks previously polymerized under high heat and pressure [174]. This results in low-porosity and a highly condensed material with superior mechanical and chemical properties when compared to conventionally processed resin [175,176]. Rapid prototyping (RP) is an automated process that creates physical models from computerized three-dimensional (3D) data [177]. Additive manufacturing techniques such as three-dimensional (3D) printing, selective laser stereolithography (SLA), selective laser melting (SLM), and selective laser sintering (SLS) are employed in RP. This technique is used in the creation of removable partial dentures. It improved the accuracy and fit of RPD frameworks [174]. Cobalt-chromium is the most commonly used material for manufacturing RPDs with SLM [174].

#### 7.2.1. Complete Denture

**Figure 3:**
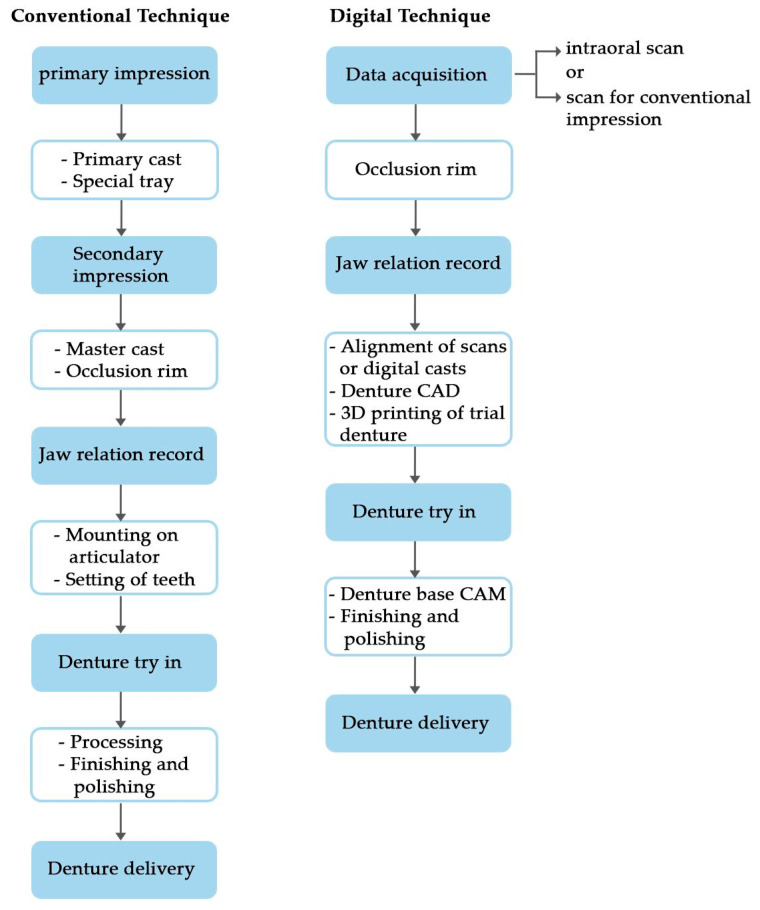
Comparison of conventional and digital workflow.

Two methods are available for creating models to fabricate dentures: conventional and intraoral digital impressions. In the traditional technique, casts are scanned using a digital scanner, and the maxillomandibular relation transfer is achieved using conventional impression and transfer techniques, the Dentca system kit, or the AvaDent system kit. The system’s computer software is employed in the laboratory to define and mark the denture borders. Following this step, the teeth are virtually set, and the denture base is milled using traditional denture resin material [173]. A study by Steinmassl et al. (2017) assessed the different clinical fabrication protocols of currently available CAD/CAM denture systems [175]. Figure 3 compares the workflow between conventional and CAD/CAM denture systems.

#### 7.2.2. Partial Denture

To create dental casts, either a conventional impression technique or a digital impression technique can be used. The conventional technique scans are cast using a digital scanner, and the removable partial denture (RPD) insertion path is digitally defined. Using this information, the shape of the framework components is a 3D design. Finally, digitally designed metal RPD frameworks are produced using rapid prototyping [173].

##### Milled Titanium Framework for RPDs

Fabricating a CP titanium framework for an implant superstructure begins with creating and scanning the framework pattern using a laboratory scanner, after which the framework is milled [72]. A one-piece, full-arch fixed prosthesis framework can be milled from a CP titanium disk to ensure enhanced fitting accuracy [73]. Sacrificial patterns for RPD frameworks are produced using CAD/CAM (milling or rapid prototyping technology), and investment-casting and finishing techniques are typically performed using conventional methods [74]. However, milling RPD frameworks from titanium disks is not cost-effective, as the bilateral RPD framework has a thin and slender shape with a delicately designed clasp and connector. Milled titanium frameworks have several drawbacks, including difficulties in cutting complicated shapes and/or undercut areas, large quantities of cutting chips being discharged, milling accuracy being compromised when cutting tools are worn, and long processing times being required.

##### Additive Manufacturing of Titanium Framework for RPDs

Recently, laser sintering and metal additive manufacturing have been employed in the production of frameworks for dental applications [77,78,79]. Compared to milling, additive manufacturing offers several advantages, such as the absence of cutting chips, the capability to produce shapes with free curves, undercuts, and hollow structures, the accuracy that is not diminished by worn cutting tools, the ability to produce many frameworks simultaneously, and a relatively low cost [78]. However, conventional additive manufacturing has one significant drawback: the particle size is often larger than 50 μm, resulting in a rough surface [79].

## 8. Comparison of Polymeric and Casting Alloys DBMs

The metals present in the casting alloy ionize while inserted in the oral cavity. The difference in alloy potentials causes metallic ions to convert to electrolytes, causing corrosion. An oxide coating develops on the surface of the alloy by absorbing oxygen, limiting further corrosion. Biofilm is a surface layer that develops on all mouth surfaces due to protein and glycoprotein precipitation from saliva [144]. Consequently, the chemical components of saliva, especially the organic components, affect the corrosive endurance of dental metals. Biofilm influences ion conversion between the alloy surface and the surrounding environment. The most prevalent is sulfide bio-film, which forms when silver or copper sulfide is formed [161,178]. These interactions between alloys and sulfur from food and beverages induce alloy discoloration and reduce further corrosive activity.

The main issue with metallic DBMs is their corrosion. Despite the creation of an oxide layer and a biofilm, corrosion in the mouth continues owing to the ongoing circulation of saliva. Melted ions react in contact with fresh saliva, and the alloy releases the new ones, causing additional corrosion. High friction forces during mastication corrode and deteriorate quicker than those exposed to low friction forces [162]. This is explained by eliminating the passive oxide layer on the alloy’s surface during mastication. The alloy wears down even more when the fresh layer is removed, and deeper alloy layers are impacted by corrosion, weakening the whole work. Non-precious dental alloys are becoming increasingly popular because of cost considerations, dental alloys, and corrosion resistance. These are predominantly Co-Cr and Ni-Cr alloys, which are much less expensive than gold [163]. According to research, the presence of sodium fluoride (Na-F) in saliva does not modify the corrosive activity of Pd alloys but accelerates the corrosion of titanium alloys [178].

Precious metals and titanium buildups had the greatest corrosion stability in artificial saliva after 1, 3, 7, 42, and 84 days. The stability of cobalt-chromium (Co-Cr) alloys was lower. The first day of Fe-Cr-Ni alloys had severe corrosive instability, but following a passive phase, these alloys demonstrated acceptable corrosive stability. The influence of saliva’s variable pH on the hardness of Co-Cr alloy has been reported. It was determined that after ninety days in a corrosive bath with a pH of 4.2, the hardness of most samples neared zero [140]. Corrosive resistance is directly related to alloy biocompatibility. As alloy disintegrates, its toxic components begin to cause harm to the body due to corrosion. Although many dental alloys lack corrosion resistance compared to titanium, this does not exclude their usage. When the poisonous level of a given element is compared to the amount that dissolves daily in the mouth due to corrosion, it is frequently necessary to wait for the whole crown to dissolve for a toxic dosage to be released. Before discontinuing standard dental alloys, a more thorough investigation of their corrosion has been proposed. Precious alloys may also be corrosive, though remarkably less than non-precious alloys [144,161].

Titanium has been the material of choice for alloy implantation because of its physical and chemical properties. Implanting non-precious alloys might expand to other economic alloys by coating non-precious metals with corrosion-resistant alloys. Investigations have been conducted on several coatings that, in addition to anticorrosive properties, have other matching ones, and so find extensive therapeutic use [179]. It has been established that there is a link between hardness and cobalt concentration since it was discovered that as cobalt (Co) content grew, so did hardness. There is also a link between chromium concentration and corrosion resistance (re-passivation potential), with an increase in chromium (Cr) concentration increasing the capacity for re-passivation [179]. The most destructive component in dental work is electrochemical corrosion. Corrosion is the accidental deterioration of metal surfaces and damage to their outer and inner layers caused by chemical or electrochemical reactions in the surrounding environment [144,171]. For electrochemical reactions, an electrolyte is required. Saliva and soft and hard tissue serve as electrolytes in the mouth. Saliva has a significant corrosive impact. As the pH factor of saliva lowers, the chloride content rises with a corrosive potential. In most alloy-electrolyte systems, corrosion is stopped at the surface by forming a surface oxide layer, which provides adequate corrosion protection. In the mouth, two protective layers form an oxide layer and a biofilm [180].

These alloys’ benefits include inexpensive casting costs, matching thermal expansion coefficients with metal, ceramic repair ceramics, and acceptable mechanical and tribological characteristics in vivo. Despite these advancements, the alloys’ flaws have not been solved when combined with inappropriate management practices, such as casting in the oxidizing zone of the flame or overheating the alloy; recurring failures may occur [141,178,181]. Several studies revealed that there is a significant metal loss in both the removable partial denture and complete denture frameworks during finishing or polishing techniques, resulting in improper contact at the tooth–clasp interface, poor fit of retentive clasp arms, and ill fitness of the denture base, and all of which affect denture retention and stability. Cleaning and smoothing of these metals after casting require specific equipment due to their extreme hardness, limiting these types of treatments in the clinics. Furthermore, machining has been frequently assigned to commercial labs that may lack standardized direct-flame casting equipment, resulting in rough and porous surfaces on frameworks [161,162,163,168]. Base metal alloys have the most significant drawback in denture retention and stability over time, followed by the risk of RPD clasp fracture and denture base perforation during adjustments owing to exterior faults and internal micro porosities. The influence of finishing and polishing on the surface roughness of cobalt-chromium (Co-Cr) castings was studied, and it was shown that contouring requires adequate smoothening procedures. This method will reduce plaque retention, enhance dental health, and boost alloy corrosion resistance. Other dental casting alloy flaws include shrinkage, porosity, inclusion, micro-cracks, and dendritic structure [163,178].

## 9. Conclusions and Future Aspects

This article comprehensively reviewed the current knowledge about denture base material’s types, properties, modifications, applications, and construction methods. Although PMMA has several benefits and gained popularity as a denture base material, it has certain limitations and cannot be classified as an ideal biomaterial for fabricating dental prostheses. Accordingly, several studies have been performed to enhance the physical and mechanical properties of PMMA via chemical modifications and mechanical reinforcement using fibers, nanofillers, and hybrid materials. However, improving one set of the material’s properties without compromising the rest of the properties remains the main challenge for researchers and clinicians in developing a modified PMMA material for denture applications.

## Data Availability

Data is available upon request from authors.

## References

[1] Hassan M., Asghar M., Din S.U., Zafar M.S. (2019). Thermoset polymethacrylate-based materials for dental applications. Materials for Biomedical Engineering.

[2] Zafar M.S. (2020). Prosthodontic Applications of Polymethyl Methacrylate (PMMA): An Update. Polymers.

[3] Anusavice K.J., Shen C., Rawls H.R. (2012). Phillips’ Science of Dental Materials.

[4] Rickman L.J., Padipatvuthikul P., Satterthwaite J.D., McCord J.F., Grey N.J., Winstanley R.B., Johnson A., George G.S., Hussain S., Welfare R. (2012). Contemporary denture base resins: Part 1. Dent. Updat..

[5] Van Noort R., Barbour M. (2014). Introduction to Dental Materials-e-Book.

[6] Khan A.A., Fareed M.A., Alshehri A.H., Aldegheishem A., Alharthi R., Saadaldin S.A., Zafar M.S. (2022). Mechanical Properties of the Modified Denture Base Materials and Polymerization Methods: A Systematic Review. Int. J. Mol. Sci..

[7] Cohen R.A. (1959). Methods and Materials Used for Artificial Teeth [Abridged].

[8] Sheejith M., Swapna C., Roshy S.N.G. (2018). Evolution of denture base material: From past to new era. IOSR J. Dent. Medic. Sci..

[9] Sheng T., Shafee M., Ariffin Z., Jaafar M. (2018). Review on poly-methyl methacrylate as denture base materials. Malays. J. Microsc..

[10] Jadhav S.S., Mahajan N., Sethuraman R. (2018). Comparative evaluation of the amount of the residual monomer in conventional and deep-frozen heat cure polymethylmethacrylate acrylic resin: An in vitro study. J. Indian Prosthodont. Soc..

[11] Singh R.D., Gautam R., Siddhartha R., Singh B.P., Chand P., Sharma V.P., Jurel S.K. (2013). High Performance Liquid Chromatographic Determination of Residual Monomer Released from Heat-Cured Acrylic Resin. An In Vivo Study. J. Prosthodont..

[12] Baemmert R.J., Lang B.R., Barco M.T., Billy E.J. (1990). Effects of denture teeth on the dimensional accuracy of acrylic resin denture bases. Int. J. Prosthodont..

[13] International Organization for Standardization (2013). Dentistry–Base Polymers–Part 1: Denture Base Polymers.

[14] Tuna S.H., Keyf F., Gumus H.O., Uzun C. (2008). The Evaluation of Water Sorption/Solubility on Various Acrylic Resins. Eur. J. Dent..

[15] Kotian R., Saini R., Madhyastha P., Srikant N. (2016). Comparative study of sorption and solubility of heat-cure and self-cure acrylic resins in different solutions. Indian J. Dent. Res..

[16] Kopperud H.M., Kleven I.S., Wellendorf H. (2011). Identification and quantification of leachable substances from polymer-based orthodontic base-plate materials. Eur. J. Orthod..

[17] LLang A., Mattie P.A., Rawis H.R. (2000). The effect of triphenylbismuth on the radiopacity and performance properties of com-pression-and injection-molded denture resins. J. Prosthodont..

[18] Barbosa D.B., de Souza R.F., Pero A.C., Marra J., Compagnoni M.A. (2007). Flexural strength of acrylic resins polymerized by different cycles. J. Appl. Oral Sci..

[19] Stafford G., Huggett R., Causton B. (1980). Fracture toughness of denture base acrylics. J. Biomed. Mater. Res..

[20] Robinson J., McCabe J. (1993). Impact strength of acrylic resin denture base materials with surface defects. Dent. Mater..

[21] Zafar M.S., Ahmed N. (2014). Nanoindentation and surface roughness profilometry of poly methyl methacrylate denture base ma-terials. Technol. Health Care.

[22] Ibrahim S.A., Lafta S.H., Hussain W.A. (2023). Effect of Reinforcing PMMA Denture Base Material by Stainless Steel 316L Wires on Flexural Strength. J. Appl. Sci. Nanotechnol..

[23] Potewiratnanond P., Ekrojanakul C., Harikul T., Kositvanich R. (2023). Wear effects between polymethyl methacrylate occlusal splints and opposing dentin surfaces during bruxism mimicking events. BDJ Open.

[24] León B.L.T., Cury A.A.D.B., Garcia R.C.M.R. (2008). Loss of residual monomer from resilient lining materials processed by different methods. Rev. Odonto Ciência.

[25] Faltermeier A., Rosentritt M., Müssig D. (2007). Acrylic removable appliances: Comparative evaluation of different postpolymerization methods. Am. J. Orthod. Dentofac. Orthop..

[26] Kawahara T., Nomura Y., Tanaka N., Teshima W., Okazaki M., Shintani H. (2004). Leachability of plasticizer and residual monomer from commercial temporary restorative resins. J. Dent..

[27] Ferracane J.L. (2006). Hygroscopic and hydrolytic effects in dental polymer networks. Dent. Mater..

[28] Santerre J., Shajii L., Leung B. (2001). Relation of Dental Composite Formulations to Their Degradation and the Release of Hydrolyzed Polymeric-Resin-Derived Products. Crit. Rev. Oral Biol. Med..

[29] Santerre J., Shajii L., Tsang H. (1999). Biodegradation of commercial dental composites by cholesterol esterase. J. Dent. Res..

[30] Finer Y., Santerre J. (2004). Salivary Esterase Activity and Its Association with the Biodegradation of Dental Composites. J. Dent. Res..

[31] Jaffer F., Finer Y., Santerre J. (2002). Interactions between resin monomers and commercial composite resins with human saliva derived esterases. Biomaterials.

[32] Lin B.A., Jaffer F., Duff M.D., Tang Y.W., Santerre J.P. (2005). Identifying enzyme activities within human saliva which are relevant to dental resin composite biodegradation. Biomaterials.

[33] Yourtee D., Smith R., Russo K., Burmaster S., Cannon J., Eick J., Kostoryz E. (2001). The stability of methacrylate biomaterials when enzyme challenged: Kinetic and systematic evaluations. J. Biomed. Mater. Res..

[34] Larsen I.B., Munksgaard E.G. (1991). Effect of human saliva on surface degradation of composite resins. Eur. J. Oral Sci..

[35] Larsen I., Freund M., Munksgaard E. (1992). Change in Surface Hardness of BisGMA/TEGDMA Polymer due to Enzymatic Action. J. Dent. Res..

[36] Iqbal Z., Zafar M.S. (2016). Role of antifungal medicaments added to tissue conditioners: A systematic review. J. Prosthodont. Res..

[37] Shaikh M.S., Alnazzawi A., Habib S.R., Lone M.A., Zafar M.S. (2021). Therapeutic Role of Nystatin Added to Tissue Conditioners for Treating Denture-Induced Stomatitis: A Systematic Review. Prosthesis.

[38] D’Ambrosio F., Santella B., Di Palo M.P., Giordano F., Giudice R.L. (2023). Characterization of the Oral Microbiome in Wearers of Fixed and Removable Implant or Non-Implant-Supported Prostheses in Healthy and Pathological Oral Conditions: A Narrative Review. Microorganisms.

[39] Chladek G., Nowak M., Pakieła W., Mertas A. (2022). Effect of *Candida albicans* Suspension on the Mechanical Properties of Denture Base Acrylic Resin. Materials.

[40] Gligorijević N., Mihajlov-Krstev T., Kostić M., Nikolić L., Stanković N., Nikolić V., Dinić A., Igić M., Bernstein N. (2022). Antimicrobial properties of silver-modified denture base resins. Nanomaterials.

[41] Farid D.A.M., Zahari N.A.H., Said Z., Ghazali M.I.M., Hao-Ern L., Zol S.M., Aldhuwayhi S., Alauddin M.S. (2022). Modification of Polymer Based Dentures on Biological Properties: Current Update, Status, and Findings. Int. J. Mol. Sci..

[42] Willershausen B., Callaway A., Ernst C.P., Stender E. (1999). The influence of oral bacteria on the surfaces of resin-based dental re-storative materials-an in vitro study. Int. Dent. J..

[43] Bettencourt A.F., Neves C.B., de Almeida M.S., Pinheiro L.M., e Oliveira S.A., Lopes L.P., Castro M.F. (2010). Biodegradation of acrylic based resins: A review. Dent. Mater..

[44] Ebadian B., Razavi M., Soleimanpour S., Mosharraf R. (2008). Evaluation of Tissue Reaction to Some Denture-base Materials: An Animal Study. J. Contemp. Dent. Pract..

[45] Boeckler A.F., Morton D., Poser S., Dette K.-E. (2008). Release of dibenzoyl peroxide from polymethyl methacrylate denture base resins: An in vitro evaluation. Dent. Mater..

[46] Munksgaard E.C. (2005). Plasticizers in denture soft-lining materials: Leaching and biodegradation. Eur. J. Oral Sci..

[47] Graham B., Jones D., Sutow E. (1991). An in vivo and in vitro Study of the Loss of Plasticizer from Soft Polymer-gel Materials. J. Dent. Res..

[48] Zissis A., Yannikakis S., Polyzois G., Harrison A. (2008). A long term study on residual monomer release from denture materials. Eur. J. Prosthodont. Restor. Dent..

[49] Kawaguchi M., Takahashi Y., Fukushima T., Habu T. (1996). Effect of light-exposure duration on the amount of leachable monomers from light-activated reline material. J. Prosthet. Dent..

[50] Lai Y.L., Chen Y.T., Lee S.Y., Shieh T.M., Hung S.L. (2004). Cytotoxic effects of dental resin liquids on primary gingival fibroblasts and periodontal ligament cells in vitro. J. Oral Rehabil..

[51] Sofou A., Tsoupi I., Karayannis M., Owall B. (2007). Determination of residual monomers released from soft lining materials with the use of HPLC. Pak. J. Anal. Environ. Chem..

[52] Brożek R., Rogalewicz R., Koczorowski R., Voelkel A. (2008). The influence of denture cleansers on the release of organic compounds from soft lining materials. J. Environ. Monit..

[53] Gonçalves T.S., de Menezes L.M., Silva L.E.A. (2008). Residual monomer of autopolymerized acrylic resin according to different ma-nipulation and polishing methods: An in situ evaluation. Angle Orthod..

[54] Urban V.M., Machado A.L., Vergani C.E., Giampaolo E.T., Pavarina A.C., de Almeida F.G., Cass Q.B. (2009). Effect of water-bath post-polymerization on the mechanical properties, degree of conversion, and leaching of residual compounds of hard chairside reline resins. Dent. Mater..

[55] Koda T., Tsuchiya H., Yamauchi M., Ohtani S., Takagi N., Kawano J. (1990). Leachability of denture-base acrylic resins in artificial saliva. Dent. Mater..

[56] Kedjarune U., Charoenworaluk N., Koontongkaew S. (1999). Release of methyl methacrylate from heat-curved and autopolymerized resins: Cytotoxicity testing related to residual monomer. Aust. Dent. J..

[57] Baker S., Brooks S., Walker D. (1988). The Release of Residual Monomeric Methyl Methacrylate from Acrylic Appliances in the Human Mouth: An Assay for Monomer in Saliva. J. Dent. Res..

[58] Azzarri M., Cortizo M., Alessandrini J. (2003). Effect of the curing conditions on the properties of an acrylic denture base resin mi-crowave-polymerised. J. Dent..

[59] Çelebi N., Yüzügüllü B., Canay Ş., Yücel Ü. (2008). Effect of polymerization methods on the residual monomer level of acrylic resin denture base polymers. Polym. Adv. Technol..

[60] Lygre H., Solheim E., Gjerdet N.R., Berg E. (1993). Leaching of organic additives from dentures in vivo. Acta Odontol. Scand..

[61] Lygre H., Klepp K.N., Solheim E., Gjerdet N.R. (1994). Leaching of additives and degradation products from cold-cured orthodontic resins. Acta Odontol. Scand..

[62] Tsuchiya H., Hoshino Y., Tajima K., Takagi N. (1994). Leaching and cytotoxicity of formaldehyde and methyl methacrylate from acrylic resin denture base materials. J. Prosthet. Dent..

[63] Hauser R., Calafat A. (2005). Phthalates and human health. Occup. Environ. Med..

[64] Pielichowski K., Njuguna J., Majka T.M. (2022). Thermal Degradation of Polymeric Materials.

[65] Sadamori S., Kotani H., Hamada T. (1992). The usage period of dentures and their residual monomer contents. J. Prosthet. Dent..

[66] Sofou A., Tsoupi I., Emmanouil J., Karayannis M. (2005). HPLC determination of residual monomers released from heat-cured acrylic resins. Anal. Bioanal. Chem..

[67] de Mello J.A.N., Braun K.O., Rached R.N., Cury A.A.D.B. (2003). Reducing the negative effects of chemical polishing in acrylic resins by use of an additional cycle of polymerization. J. Prosthet. Dent..

[68] Lefebvre C.A., Schuster G.S., Marr J.C., Knoernschild K.L. (1995). The effect of pH on the cytotoxicity of eluates from denture base resins. Int. J. Prosthodont..

[69] Tsuchiya H., Hoshino Y., Kato H., Takagi N. (1993). Flow injection analysis of formaldehyde leached from denture-base acrylic resins. J. Dent..

[70] Keyf F., Keyf A.I. (1998). Harmful effects of methylmethacrylate and formaldehyde from acrylic resin denture base materials. Saudi Dent. J..

[71] Hashimoto Y., Kawaguchi M., Miyazaki K., Nakamura M. (2003). Estrogenic activity of tissue conditioners in vitro. Dent. Mater..

[72] Jorge J.H., Giampaolo E.T., Machado A.L., Vergani C.E. (2003). Cytotoxicity of denture base acrylic resins: A literature review. J. Prosthet. Dent..

[73] Golbidi F., Asghari G. (2009). The level of residual monomer in acrylic denture base materials. Res. J. Biol. Sci..

[74] Giunta J., Zablotsky N. (1976). Allergic stomatitis caused by self-polymerizing resin. Oral Surg. Oral Med. Oral Pathol..

[75] Huang F.-M., Tai K.-W., Hu C.-C., Chang Y.-C. (2001). Cytotoxic effects of denture base materials on a permanent human oral epi-thelial cell line and on primary human oral fibroblasts in vitro. Int. J. Prosthodont..

[76] Schuster G.S., Lefebvre C.A., Dirksen T.R., Knoernschild K.L., Caughman G.B. (1995). Relationships between denture base resin cytotoxicity and cell lipid metabolism. Int. J. Prosthodont..

[77] Yang H.-W., Chou L.S.-S., Chou M.-Y., Chang Y.-C. (2003). Assessment of genetic damage by methyl methacrylate employing in vitro mammalian test system. Biomaterials.

[78] Jorge J.H., Giampaolo E.T., Vergani C.E., Machado A.L., Pavarina A.C., Carlos I.Z. (2006). Effect of post-polymerization heat treat-ments on the cytotoxicity of two denture base acrylic resins. J. Appl. Oral Sci..

[79] Schweikl H., Schmalz G., Spruss T. (2001). The induction of micronuclei in vitro by unpolymerized resin monomers. J. Dent. Res..

[80] Schweikl H., Spagnuolo G., Schmalz G. (2006). Genetic and Cellular Toxicology of Dental Resin Monomers. J. Dent. Res..

[81] Dixon D.L., Breeding L.C., Ekstrand K.G. (1992). Linear dimensional variability of three denture base resins after processing and in water storage. J. Prosthet. Dent..

[82] Cucci A.L.M., Rached R.N., Giampaolo E.T., Vergani C.E. (1999). Tensile bond strengths of hard chairside reline resins as influenced by water storage. J. Oral Rehabil..

[83] Anusavice K.J. (2003). Philip’s Science of Dental Materials.

[84] Mese A., Guzel K.G. (2008). Effect of storage duration on the hardness and tensile bond strength of silicone- and acrylic resin-based resilient denture liners to a processed denture base acrylic resin. J. Prosthet. Dent..

[85] Dixon D.L., Ekstrand K.G., Breeding L.C. (1991). The transverse strengths of three denture base resins. J. Prosthet. Dent..

[86] Takahashi Y., Chai J., Kawaguchi M. (1999). Equilibrium strengths of denture polymers subjected to long-term water immersion. Int. J. Prosthodont..

[87] Craig R. (2002). Scope and History of Restorative Materials, Restorative Dental Materials.

[88] Muraoka G., Takahashi H., Hayakawa I. (2003). Effects of cyclic loading on viscoelastic properties of soft lining materials. Dent. Mater. J..

[89] Jepson N.J., McGill J.T., McCabe J.F. (2000). Influence of dietary simulating solvents on the viscoelasticity of temporary soft lining materials. J. Prosthet. Dent..

[90] Ferracane J., Berge H. (1995). Fracture Toughness of Experimental Dental Composites Aged in Ethanol. J. Dent. Res..

[91] Al-Bakri I., Harty D., Al-Omari W., Swain M., Chrzanowski W., Ellakwa A. (2014). Surface characteristics and microbial adherence ability of modified polymethylmethacrylate by fluoridated glass fillers. Aust. Dent. J..

[92] Tsutsumi C., Takakuda K., Wakabayashi N. (2016). Reduction of Candida biofilm adhesion by incorporation of prereacted glass ionomer filler in denture base resin. J. Dent..

[93] Sawada T., Sawada T., Kumasaka T., Hamada N., Shibata T., Nonami T., Kimoto K. (2014). Self-cleaning elects of acrylic F. Gerodontology.

[94] Shibata T., Hamada N., Kimoto K., Sawada T., Sawada T., Kumada H., Umemoto T., Toyoda M. (2007). Antifungal e_ect of acrylic resin containing apatite-coated TiO_2_ photocatalyst. Dent. Mater. J..

[95] Akihiro Yoshihara D., Sakuma P.S., Kobayashi P.S., Miyazaki P.H. (2001). Antimicrobial effect of fluoride mouthrinse on mutans streptococci and lactobacilli in saliva. Pediatr. Dent..

[96] Casemiro L.A., Martins C.H.G., Pires-De-Souza F.d.C.P., Panzeri H. (2008). Antimicrobial and mechanical properties of acrylic resins with incorporated silver-zinc zeolite—Part I. Gerodontology.

[97] Flores J.C., Garcia R., Villanueva G., Acosta-Torres L. (2020). Antimicrobial poly (methyl methacrylate) with silver nanoparticles for dentistry: A systematic review. Appl. Sci..

[98] Kurt A., Erkose-Genc G., Uzun M., Emrence Z., Ustek D., Isik-Ozkol G. (2017). The antifungal activity and cytotoxicity of silver containing denture base material. Niger. J. Clin. Pract..

[99] Acosta-Torres L.S., Mendieta I., Nunez-Anita R.E., Cajero-Juarez M., Castano V.M. (2012). Cytocompatible antifungal acrylic resin containing silver nanoparticles for dentures. Int. J. Nanomed..

[100] Monteiro D.R., Gorup L.F., Takamiya A.S., de Camargo E.R., Filho A.C.R., Barbosa D.B. (2012). Silver Distribution and Release from an Antimicrobial Denture Base Resin Containing Silver Colloidal Nanoparticles. J. Prosthodont..

[101] Mangal U., Kim J.-Y., Seo J.-Y., Kwon J.-S., Choi S.-H. (2019). Novel Poly(Methyl Methacrylate) Containing Nanodiamond to Improve the Mechanical Properties and Fungal Resistance. Materials.

[102] Lee J.-H., El-Fiqi A., Jo J.-K., Kim D.-A., Kim S.-C., Jun S.-K., Kim H.-W., Lee H.-H. (2016). Development of long-term antimicrobial poly(methyl methacrylate) by incorporating mesoporous silica nanocarriers. Dent. Mater..

[103] Gad M.M., Al-Thobity A.M., Fouda S.M., Näpänkangas R., Raustia A. (2020). Flexural and Surface Properties of PMMA Denture Base Material Modified with Thymoquinone as an Antifungal Agent. J. Prosthodont..

[104] Makvandi P., Jamaledin R., Jabbari M., Nikfarjam N., Borzacchiello A. (2018). Antibacterial quaternary ammonium compounds in dental materials: A systematic review. Dent. Mater..

[105] Makvandi P., Ghaemy M., Mohseni M. (2016). Synthesis and characterization of photo-curable bis-quaternary ammonium di-methacrylate with antimicrobial activity for dental restoration materials. Eur. Polym. J..

[106] Makvandi P., Ghaemy M., Ghadiri A., Mohseni M. (2015). Photocurable, antimicrobial quaternary ammonium–modified nano-silica. J. Dent. Res..

[107] Pesci-Bardon C., Fosse T., Serre D., Madinier I. (2006). In vitro antiseptic properties of an ammonium compound combined with denture base acrylic resin. Gerodontology.

[108] Pesci-Bardon C., Fosse T., Madinier I., Serre D. (2004). Flores denture acrylic resin. Lett. Appl. Microbiol..

[109] Delfi M., Ghomi M., Zarrabi A., Mohammadinejad R., Taraghdari Z.B., Ashrafizadeh M., Zare E.N., Agarwal T., Padil V.V.T., Mokhtari B. (2020). Functionalization of Polymers and Nanomaterials for Biomedical Applications: Antimicrobial Platforms and Drug Carriers. Prosthesis.

[110] Mai H.-N., Kim D.-Y., Hyun D.C., Park J.H., Lee S.M., Lee D. (2019). A New Antibacterial Agent-Releasing Polydimethylsiloxane Coating for Polymethyl Methacrylate Dental Restorations. J. Clin. Med..

[111] Lee J.-H., Jo J.-K., Kim D.-A., Patel K.D., Kim H.-W., Lee H.-H. (2018). Nano-graphene oxide incorporated into PMMA resin to prevent microbial adhesion. Dent. Mater..

[112] Ratanajanchai M., Kanchanavasita W., Suputtamongkol K., Wonglamsam A., Thamapipol S., Sae-Khow O. (2020). Heat-cured poly(methyl methacrylate) resin incorporated with di_erent food preservatives as an anti-microbial denture base material. J. Dent. Sci..

[113] Lassila L., Vallittu P. (2001). Denture base polymer Alldent Sinomer®: Mechanical properties, water sorption and release of residual compounds. J. Oral Rehabil..

[114] Najeeb S., Zafar M.S., Khurshid Z., Siddiqui F. (2016). Applications of polyetheretherketone (PEEK) in oral implantology and pros-thodontics. J. Prosthodont. Res..

[115] Zol S.M., Alauddin M.S., Said Z., Ghazali M.I.M., Hao-Ern L., Farid D.A.M., Zahari N.A.F.H., Al-Khadim A.H.A., Aziz A.H.A. (2023). Description of Poly (aryl-ether-ketone) Materials (PAEKs), Polyetheretherketone (PEEK) and Polyetherketon-eketone (PEKK) for Application as a Dental Material: A Materials Science Review. Polymers.

[116] Silva L.S., Bento V.A.A., Brunetto J.L., Pesqueira A.A. (2023). Polyetheretherketone materials for removable partial denture frameworks: An integrative review. Gen. Dent..

[117] Alqurashi H., Khurshid Z., Syed A.U.Y., Habib S.R., Rokaya D., Zafar M.S. (2021). Polyetherketoneketone (PEKK): An emerging biomaterial for oral implants and dental prostheses. J. Adv. Res..

[118] Consani R.L.X., Domitti S.S., Barbosa C.M.R., Consani S. (2002). Effect of commercial acrylic resins on dimensional accuracy of the maxillary denture base. Braz. Dent. J..

[119] Cruzeiro M.T.R., Moraes F.A., Kaizer M.R., Moreira M.L., Zhang Y., Moraes R.R., Cava S.S. (2017). Functionalized pink Al_2_O_3_: Mn pigments applied in prosthetic dentistry. J. Prosthet. Dent..

[120] Alrahlah A., Fouad H., Hashem M., Niazy A.A., AlBadah A. (2018). Titanium oxide (TiO_2_)/polymethylmethacrylate (PMMA) denture base nanocomposites: Mechanical, vis-coelastic and antibacterial behavior. Materials.

[121] Alamgir, Mallick A., Nayak G.C., Tiwari S.K. (2019). Development of PMMA/TiO_2_ nanocomposites as excellent dental materials. J. Mech. Sci. Technol..

[122] Grant A., Atkinson H. (1971). Comparison between dimensional accuracy of dentures produced with pour-type resin and with heat-processed materials. J. Prosthet. Dent..

[123] Nogueira S.S., Ogle R.E., Davis E.L. (1999). Comparison of accuracy between compression- and injection-molded complete dentures. J. Prosthet. Dent..

[124] Tandon R., Gupta S., Agarwal S.K. (2010). Denture base materials: From past to future. Indian J. Dent. Sci..

[125] Sakaguchi R.L., Powers J.M. (2012). Craig’s Restorative Dental Materials-e-Book.

[126] Phoenix R.D., Mansueto M.A., Ackerman N.A., Jones R.E. (2004). Evaluation of Mechanical and Thermal Properties of Commonly Used Denture Base Resins. J. Prosthodont..

[127] Kj A. (2003). Phillips’ Science of Dental Materials.

[128] John J., Gangadhar S.A., Shah I. (2001). Flexural strength of heat-polymerized polymethyl methacrylate denture resin reinforced with glass, aramid, or nylon fibers. J. Prosthet. Dent..

[129] Lee C.-J., Bok S.-B., Bae J.-Y., Lee H.-H. (2010). Comparative adaptation accuracy of acrylic denture bases evaluated by two different methods. Dent. Mater. J..

[130] Becker C.M., Smith D.E., Nicholls J.I. (1977). The comparison of denture-base processing techniques. Part II. Dimensional changes due to processing. J. Prosthet. Dent..

[131] Fujisawa M., Adachi K., Tsuruta S., Ishibashi K. (2004). A procedure for fitting a fixed partial denture to an existing removable partial denture. J. Prosthet. Dent..

[132] Garfunkel E. (1983). Evaluation of dimensional changes in complete dentures processed by injection-pressing and the pack-and-press technique. J. Prosthet. Dent..

[133] Keenan P.L., Radford D.R., Clark R.K. (2003). Dimensional change in complete dentures fabricated by injection molding and mi-crowave processing. J. Prosthet. Dent..

[134] Huggett R., Zissis A., Harrison A., Dennis A. (1992). Dimensional accuracy and stability of acrylic resin denture bases. J. Prosthet. Dent..

[135] Consani R.L.X., Domitti S.S., Mesquita M.F., Consani S. (2004). Effect of packing types on the dimensional accuracy of denture base resin cured by the conventional cycle in relation to post-pressing times. Braz. Dent. J..

[136] Memon M.S., Yunus N., Razak A.A.A., Memon M., Yunus N., Razak A. (2001). Some mechanical properties of a highly cross-linked, microwave-polymerized, injection-molded denture base polymer. Int. J. Prosthodont..

[137] Strohaver R.A. (1989). Comparison of changes in vertical dimension between compression and injection molded complete dentures. J. Prosthet. Dent..

[138] Polat T.N., Karacaer Ö., Tezvergil A., Lassila L.V.J., Vallittu P.K. (2003). Water Sorption, Solubility and Dimensional Changes of Denture Base Polymers Reinforced with Short Glass Fibers. J. Biomater. Appl..

[139] Young B.C. (2010). A Comparison of Polymeric Denture Base Materials.

[140] Donovan T.E., Cho G.C. (2003). Esthetic considerations with removable partial dentures. J. Calif. Dent. Assoc..

[141] Ono T., Kita S., Nokubi T. (2004). Dimensional accuracy of acrylic resin maxillary denture base polymerized by a new injection pressing method. Dent. Mater. J..

[142] Rejab L.T. (2008). The Effect of the Thermopress Curing Technique on the Water Sorption and Solubility of the Cold and Heat–Cured Acrylic Resins. Al-Rafidain Dent. J..

[143] Parvizi A., Lindquist T., Schneider R., Williamson D., Boyer D., Dawson D.V. (2004). Comparison of the dimensional accuracy of injection-molded denture base materials to that of conventional pressure-pack acrylic resin. J. Prosthodont. Implant. Esthet. Reconstr. Dent..

[144] Wataha J.C., Messer R.L. (2004). Casting alloys. Dent. Clin..

[145] Gad M.M., Fouda S.M., Al-Harbi F.A., Näpänkangas R., Raustia A. (2017). PMMA denture base material enhancement: A review of fiber, filler, and nanofiller addition. Int. J. Nanomed..

[146] Garcia A.A.M.N., Sugio C.Y.C., de Azevedo-Silva L.J., Gomes A.C.G., Batista A.U.D., Porto V.C., Soares S., Neppelenbroek K.H. (2021). Nanoparticle-modified PMMA to prevent denture stomatitis: A systematic review. Arch. Microbiol..

[147] Fouda S.M., Gad M.M., Ellakany P., Al Ghamdi M.A., Khan S.Q., Akhtar S., Ali M.S., Al-Harbi F.A. (2022). Flexural Properties, Impact Strength, and Hardness of Nanodiamond-Modified PMMA Denture Base Resin. Int. J. Biomater..

[148] An J., Ding N., Zhang Z. (2022). Mechanical and antibacterial properties of polymethyl methacrylate modified with zinc dimethacrylate. J. Prosthet. Dent..

[149] Correa S., Matamala L., González J.P., de la Fuente M., Miranda H., Olivares B., Maureira M., Agüero A., Gómez L., Lee X. (2023). Development of novel antimicrobial acrylic denture modified with copper nanoparticles. J. Prosthodont. Res..

[150] Tomar P., Gope P. (2023). Impact Strength Enhancement of PMMA base Denture Material by Fibre Addition. Int. J. Mater. Manuf. Sustain. Technol..

[151] Elmwafy D., Abdelghany A. (2022). Evaluation of flexural strength, microhardness and color stability of polyamide and heat cured acrylic resins modified with titania nanorods. Egypt. Dent. J..

[152] Kowalski R., Kozak M., Sobolewska E. (2023). Contemporary hybrid acrylic materials and modern thermoplastics in the manufacture of dental prostheses. Pomeranian J. Life Sci..

[153] Nabhan A., Taha M., Ghazaly N.M. (2023). Filler loading effect of Al_2_O_3_/TiO_2_ nanoparticles on physical and mechanical characteristics of dental base composite (PMMA). Polym. Test..

[154] Díez-Pascual A.M. (2022). PMMA-Based Nanocomposites for Odontology Applications: A State-of-the-Art. Int. J. Mol. Sci..

[155] Safi I.N. (2014). Evaluation the effect of nano-fillers (TiO_2_, Al_2_O_3_, SiO_2_) addition on glass transition temperature, E-Moudulus and coefficient of thermal expansion of acrylic denture base material. J. Baghdad Coll. Dent..

[156] Abdulkareem M.M., Hatim N.A. (2015). Evaluation the biological effect of adding aluminum oxide, silver nanoparticles into micro-wave treated PMMA powder. Int. J. Enhanc. Res. Sci. Technol. Eng..

[157] Alla R., Raghavendra K., Vyas R., Konakanchi A. (2015). Conventional and contemporary polymers for the fabrication of denture prosthesis: Part I–overview, composition and properties. Int. J. Appl. Dent. Sci..

[158] Engelmeier R.L. (2003). The history and development of posterior denture teeth—Introduction, part I. J. Prosthodont..

[159] Bathala L., Majeti V., Rachuri N., Singh N., Gedela S. (2019). The Role of Polyether Ether Ketone (Peek) in Dentistry—A Review. J. Med. Life.

[160] Muhsin S.A., Hatton P.V., Johnson A., Sereno N., Wood D.J. (2019). Determination of Polyetheretherketone (PEEK) mechanical properties as a denture material. Saudi Dent. J..

[161] Silva J.W.J., Sousa L.L., Nakazato R.Z., Codaro E.N., de Felipe H. (2011). Electrochemical and Microstructural Study of Ni-Cr-Mo Alloys Used in Dental Prostheses. Mater. Sci. Appl..

[162] Wataha J.C. (2000). Biocompatibility of dental casting alloys: A review. J. Prosthet. Dent..

[163] Wataha J.C., O’Dell N.L., Singh B.B., Ghazi M., Whitford G.M., Lockwood P.E. (2001). Relating nickel-induced tissue inflammation to nickel release in vivo. J. Biomed. Mater. Res..

[164] Rokaya D., Bohara S., Srimaneepong V., Kongkiatkamon S., Khurshid Z., Heboyan A., Zafar M.S., Sapkota J. (2022). Metallic bio-Materials for Medical and Dental Prosthetic Applications, Functional Biomaterials: Drug Delivery and Biomedical Applications.

[165] Özen J., Ural A.U., Dalkiz M., Beydemir B. (2005). Influence of dental alloys and an all-ceramic material on cell viability and interleu-kin-1beta release in a three-dimensional cell culture model. Turk. J. Med. Sci..

[166] Alqutaibi A.Y., Alharbi A.F., Alharbi A.M., Karbouji G.A., Dagharire E.Y., Aboalrejal A. (2022). Pretreatment expectations and posttreatment satisfaction with different prosthodontic treatments in a Saudi population. Saudi J. Oral Sci..

[167] Alqutaibi A.Y. (2020). A within-subject comparison of the conventional clasp-retained with attachment-retained removable partial dentures. J. Taibah Univ. Med. Sci..

[168] Wulfes H. (2004). Precision Milling and Partial Denture Constructions.

[169] Craig O.B. (1992). Dental Materials: Properties and Manipulation.

[170] Reclaru L., Lüthy H., Eschler P.-Y., Blatter A., Susz C. (2005). Corrosion behaviour of cobalt–chromium dental alloys doped with precious metals. Biomaterials.

[171] Wataha J.C. (2002). Alloys for prosthodontic restorations. J. Prosthet. Dent..

[172] Iacoban S., Bolat G., Munteanu C., Cailean D., Trinca L., Mareci D. (2015). A comparative study on the corrosion behaviour of CoCr and NiCr dental alloys in saline medium. Rev. Roum. Chim..

[173] Bilgin M.S., Baytaroğlu E.N., Erdem A., Dilber E. (2016). A review of computer-aided design/computer-aided manufacture techniques for removable denture fabrication. Eur. J. Dent..

[174] Ahmed N., Abbasi M.S., Haider S., Ahmed N., Habib S.R., Altamash S., Zafar M.S., Alam M.K. (2021). Fit Accuracy of Removable Partial Denture Frameworks Fabricated with CAD/CAM, Rapid Prototyping, and Conventional Techniques: A Systematic Review. BioMed Res. Int..

[175] Steinmassl P.-A., Klaunzer F., Steinmassl O., Dumfahrt H., Grunert I. (2017). Evaluation of Currently Available CAD/CAM Denture Systems. Int. J. Prosthodont..

[176] Limírio J.P.J.D.O., Gomes J.M.D.L., Rezende M.C.R.A., Lemos C.A.A., Rosa C.D.D.R.D., Pellizzer E.P. (2021). Mechanical properties of polymethyl methacrylate as a denture base: Conventional versus CAD-CAM resin—A systematic review and meta-analysis of in vitro studies. J. Prosthet. Dent..

[177] Inokoshi M., Kanazawa M., Minakuchi S. (2012). Evaluation of a complete denture trial method applying rapid prototyping. Dent. Mater. J..

[178] Bezzon O.L., Pedrazzi H., Zaniquelli O., da Silva T.B.C. (2004). Effect of casting technique on surface roughness and consequent mass loss after polishing of NiCr and CoCr base metal alloys: A comparative study with titanium. J. Prosthet. Dent..

[179] Dobrzański L. (2011). Influence of Cr and Co on hardness and corrosion resistance CoCrMo alloys used on dentures. J. Achiev. Mater. Manuf. Eng..

[180] Eliaz N. (2019). Corrosion of Metallic Biomaterials: A Review. Materials.

[181] Joias R.M., Tango R.N., de Araujo J.E.J., de Araujo M.A.J., Saavedra G.D.S.F.A., de Arruda Paes-Junior T.J., Kimpara E.T. (2008). Shear bond strength of a ceramic to Co-Cr alloys. J. Prosthet. Dent..

